# Use of a partial humeral head resurfacing system for management of an osseous mechanical block to glenohumeral joint range of movement secondary to proximal humeral fracture malunion

**DOI:** 10.4103/0973-6042.80465

**Published:** 2011

**Authors:** Kyriacos Eleftheriou, Nawfal Al-Hadithy, Vinay Joshi, Daniel Rossouw

**Affiliations:** Department of Trauma and Orthopaedics, Barnet General Hospital, Wellhouse Lane, Town Centre, Barnet EN5 3DJ; 1 Consultant Orthopaedic and Shoulder Surgeon, Barnet General Hospital, Wellhouse Lane, Town Centre, Barnet EN5 3DJ

**Keywords:** Shoulder HemiCAP malunion humerus fracture

## Abstract

Malunion of proximal humeral fractures can lead to a severely impaired shoulder function. Loss of motion is often the main issue in patients and can be secondary to osseous, soft-tissue as well as neurological damage to the shoulder. Malunion of the articular surface of the humeral head can lead to pain, chronic degenerative changes secondary to joint incongruity and mechanical block to the range of movement. A 46-year-old otherwise healthy male chef presented with malunion and collapse of his previous plate fixation for a four-part proximal humerus fracture. We describe the first documented case of the use of a focal resurfacing system for dealing with such an osseous mechanical block in the presence of an otherwise preserved articular surface in a high-demand patient. HemiCAP can be successfully used in proximal humeral fracture malunion where there is a localized cartilage defect, allowing restoration of joint congruity while preserving the bone stock.

## INTRODUCTION

Malunion of proximal humeral fractures, especially the three- and four-part types, can lead to severe functional impairment due to deformity, articular incongruity, avascular necrosis, posttraumatic arthritis as well as associated soft tissue and neurological injury. In the absence of avascular necrosis and with a relatively preserved articular surface, a reconstruction procedure rather than a head sacrificing procedure may be preferable if anatomic restoration is possible. This is challenging, however, with a high complication rate and variable outcome results.[1]

**Figure 1:**
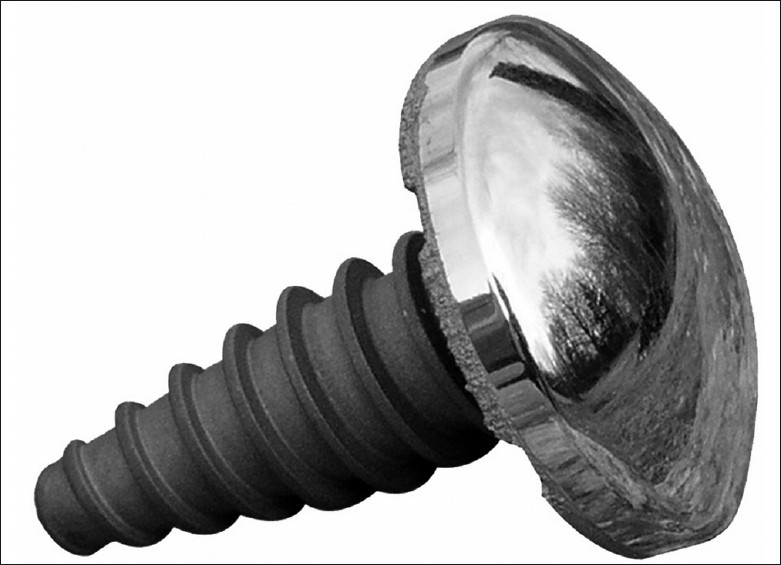
The HemiCAP implant

The HemiCAP system (Arthosurface, Franklin, MA, USA) allows a more focal resurfacing of an articular surface and is indicated in the management of humeral head osteoarthritis, avascular necrosis as well as management of local chondral defects and other isolated defects such as Hill-Sachs lesions restoring articular congruity and preserving bone stock. The system provides instruments to map and prepare the focal damaged area to allow implantation of a cobalt–chrome and titanium implant [Figure 1] that precisely aligns the contours of the articular surface and restores a smooth articular surface at the area of the defect using a range of implants of varying diameters and curvatures.

The HemiCAP system has been used successfully in the correction of Hill-Sachs lesion for recurrent anterior shoulder instability.[2] However, we describe the only documented case in managing patients with a focal osseous mechanical block to glenohumeral range of movement following malunion in the presence of an otherwise preserved articular surface in a high-demand patient.

## CASE REPORT

**Figure 2:**
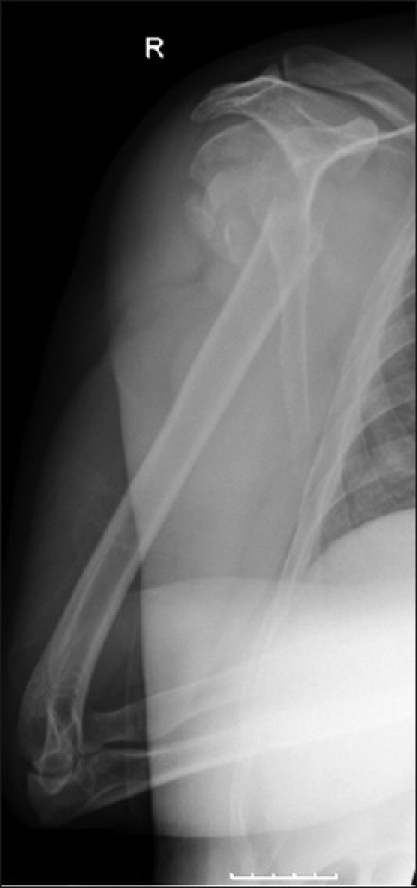
Original injury

**Figure 3:**
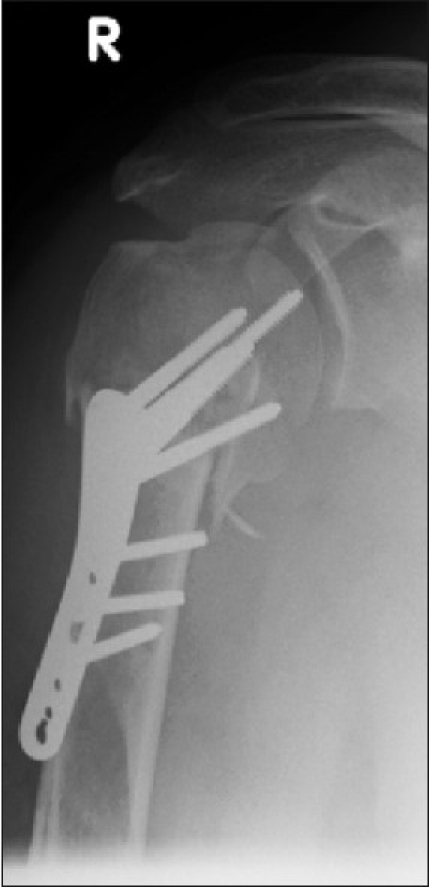
Immediate postoperative fixation

**Figure 4:**
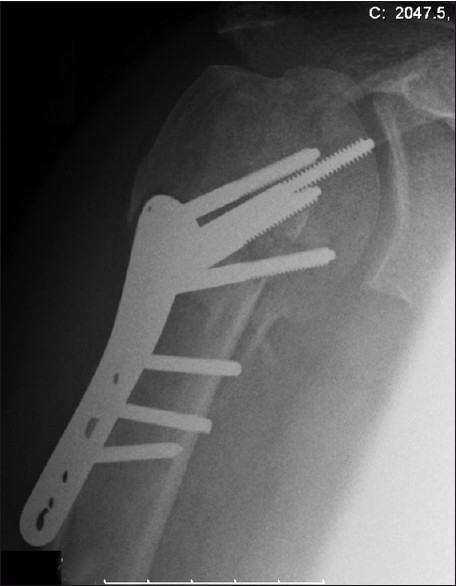
Advanced collapse at 6 weeks. The protruding screw was promptly removed

**Figure 5:**
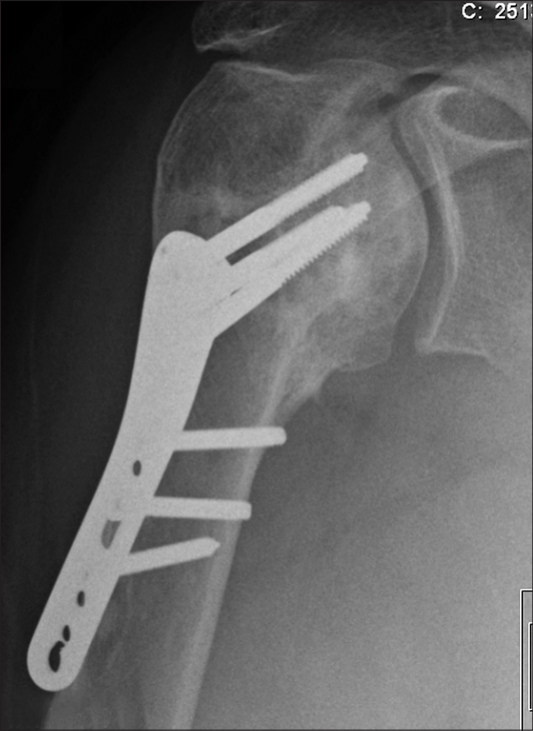
Malunion at 9 months

An active 46-year-old right hand-dominant nonsmoking healthy male chef presented with a four-part proximal humeral fracture following a motorcycling accident [Figure 2]. In view of his age, he underwent open reduction and internal fixation of the fracture with a proximal humeral locking plate [Figure 3]. After an uneventful immediate postoperative recovery, his functional improvement was limited and his range of movement and pain symptoms deteriorated over the subsequent 6 months despite intensive rehabilitation. Serial radiographs revealed a progressive collapse of the articular head part. This lead to protrusion of one of the screws into the articular region 6 weeks postoperatively [Figure 4], which was promptly removed. Over the subsequent months, his fracture continued to collapse. Radiographs at 9 months showed malunion of the fracture, with a bony spur present at the superior part of the head where the collapse segment went on to malunion [Figure 5]. On examination, the patient had 40° forward flexion, 40° abduction and 10° external rotation, with a painful mechanical block to further movement. His rotator cuff appeared clinically intact, with adequate strength otherwise and no ligamentous laxity or instability. The neurovascular exam was normal.

We felt that the bony spur was the main cause of his limited function. Given his high-demand requirement and otherwise normal-appearing articular surface that was not significantly malrotated, we elected to perform a joint-preserving procedure. Excision of the prominent bony region was considered over a restorative osteotomy; the former, however, would leave an exposed area of bone that could go on to cause pain, with the risk of recurrence of the mechanical block due to bony outgrowth. We, therefore, opted to focally resurface the area using the HemiCAP system.

### Procedure

**Figure 6:**
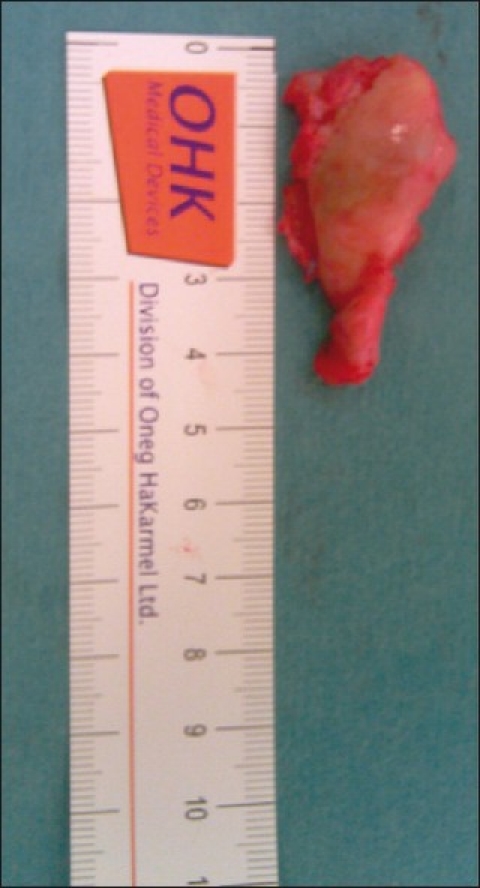
Excised bony spur

Under general anesthesia with an interscalene block and the patient in the beach chair position, a standard anterior deltopectoral approach was used through the old incision. The previous fixation plate and screws were successfully removed first and the malunion of the fracture and the presence of an intact rotator cuff were confirmed. The subdeltoid and subacromial adhesions were then released and the subscapularis and the associated capsule were incised at the lesser tuberosity and lifted off to expose the humeral head. Adequate release of the soft tissues with retraction allowed the bony spur to be easily visualised and brought into the operating field by extension and external rotation. This was removed using a fine saw [Figure 6].

**Figure 7:**
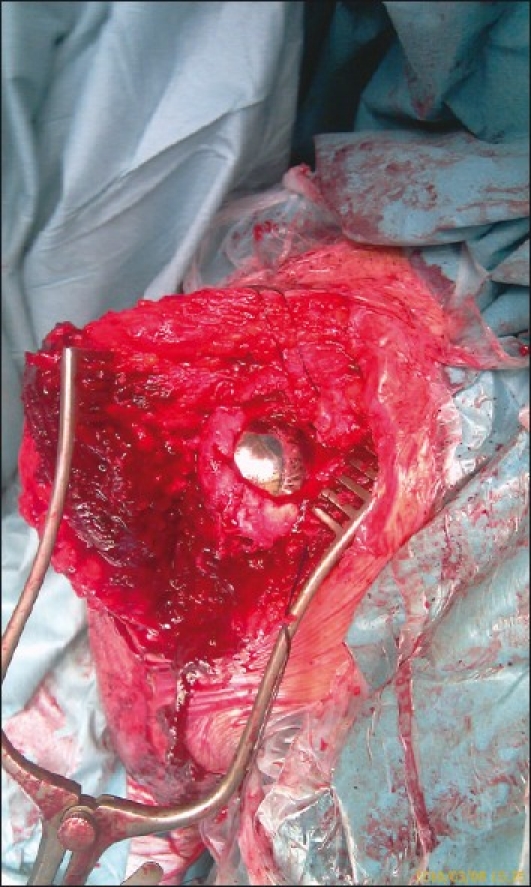
HemiCAP inserted

A guidewire was then placed central to the defect and perpendicular to the articular surface with a drill guide used to demarcate the area of concern and allowing measurement of the appropriate articular component diameter (25 mm) required to resurface the defect. A cannulated drill was then used in order to prepare the head to receive the headless titanium post. A trial Cap was then used to confirm that the post was at a correct depth so that the final prosthetic surface would be at the same level as the articular surface. The instrumentation provides a contact probe that maps the surface curvature in two planes, obtaining offsets at four indexing points. A sizing card was then used to select the appropriate articular component. An appropriate surface reamer based on the offset measurements was then used to prepare the exposed articular surface. A trial was then used to confirm the fit, ensuring that this was congruent with the edge of the surrounding humeral surface. The appropriate articular component was then placed in its post [Figure 7], restoring a smooth humeral surface at the area where the bony spur was previously present. A full and stable range of movement with no impingement or mechanical block was possible intraoperatively. The incised capsule and subscapularis were then repaired.

### Postoperative course and rehabilitation

Postoperative radiographs demonstrated adequate restoration of the humeral head articular surface at the area where the bony spur was removed. The patient was discharged on the first postoperative day and had no complications.

The patient was placed in a sling for comfort and early passive and active assisted range of movement was encouraged from the first postoperative day to minimize stiffness. Shoulder rehabilitation progressed from passive range of motion, active assisted to active shoulder movements, with a focus on improving proprioception, stability and power of both the scapula-thoracic and the gleno-humeral joints.

**Figure 8:**
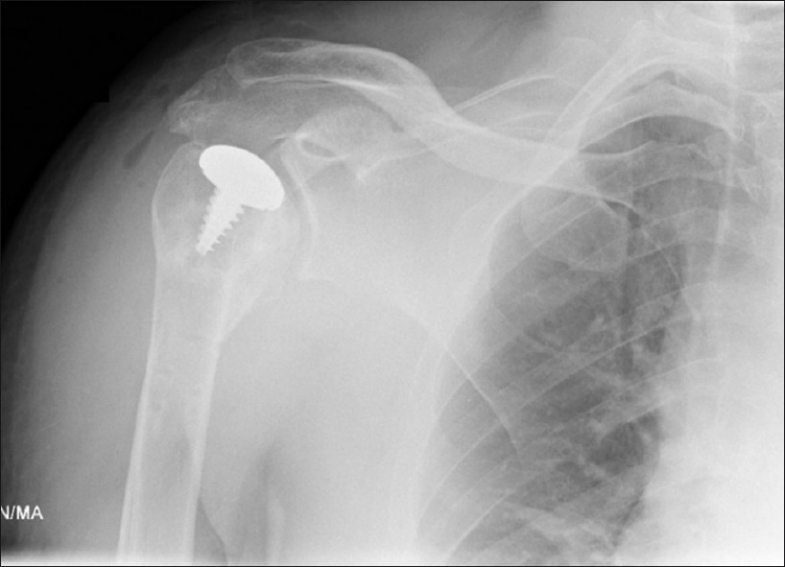
Check radiograph postoperatively

At the 6-month follow-up, an examination revealed 140° forward flexion, 120° abduction and 35° external rotation, and the patient was pain-free. The 12-month follow-up revealed 150° forward flexion, 120° abduction and 35° external rotation, and the patient was pain-free. He had gone back to work full time and had adequate function of his arm. Repeat radiographic assessment showed no loosening or displacement of the prosthesis, no new bony outgrowth and no changes to the opposing glenoid articular surface [Figure 8]. His postoperative Oxford Shoulder Score was 31 (preoperative, 15).

## DISCUSSION

Malunion of the proximal humeral fractures can result from conservative management of the injury, inadequate reduction at fixation or postoperative loss of an adequate reduction. It can lead to severe functional impairment, and its management is difficult and depends on the resulting deformity, the condition of the articular surface, soft tissues as well as patient characteristics.[3] Surgical options include arthrodesis, corrective restorative osteotomy or an arthroplasty-type procedure (hemiarthroplasty, total/reverse shoulder replacement or standard resurfacing).[1] The data on the results of the various surgical options is both limited and restricted by the variability of the resulting deformity and patient characteristics. In general, however, outcomes are generally unsatisfactory, with a significant complication rate,[4] and prevention of malunion is therefore preferable at the initial management of the acute fracture.

In our high-demand patient, the problem was relatively localized and a more limited procedure to either a corrective osteotomy or a standard resurfacing was preferable. We felt that an isolated excision osteotomy of the bony spur would not have been ideal, in that it would be difficult to restore a smooth continuous humeral head surface and would give the possibility of recurrence due to bony outgrowth.

The role of routine preoperative computerized tomography (CT) scanning has been discussed and is controversial. Bernstein *et al*.[5] concluded that proximal humeral fractures could be classified and managed with radiographs alone, which conflicted with Jurik *et al*.,[6] who recommended the routine use of CT in proximal humerus fractures. In this case, we had the benefit of serial radiographs and felt that a CT was not required.

We therefore suggest another indication of the HemiCAP focal resurfacing (or similar) system that can provide a good solution in certain cases of proximal humeral fracture malunion, where there is a localized surface problem with an otherwise relatively preserved articular surface, allowing restoration of a smooth continuous surface while preserving bone stock.
